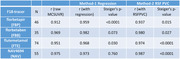# Accounting for White Matter Uptake Improves Between‐Tracer Agreement in Amyloid PET

**DOI:** 10.1002/alz.090045

**Published:** 2025-01-09

**Authors:** Yinghua Chen, Hillary D. Protas, Ji Luo, Shan Li, Javad Sohankar, Valentina Ghisays, Wendy Lee, Teresa Wu, Eric M. Reiman, Kewei Chen, Yi Su

**Affiliations:** ^1^ Banner Alzheimer's Institute, Phoenix, AZ USA; ^2^ Arizona Alzheimer's Consortium, Phoenix, AZ USA; ^3^ ASU‐Mayo Center for Innovative Imaging, Tempe, AZ USA; ^4^ School of Computing and Augmented Intelligence, Arizona State University, Tempe, AZ USA; ^5^ Biodesign Institute, Arizona State University, Tempe, AZ USA; ^6^ Department of Psychiatry, University of Arizona, Phoenix, AZ USA; ^7^ Department of Neurology College of Medicine‐Phoenix, University of Arizona, Phoenix, AZ USA; ^8^ School of Mathematics and Statistical Sciences, College of Health Solutions, Arizona State University, Tempe, AZ USA; ^9^ Arizona State University, Tempe, AZ USA; ^10^ University of Arizona College of Medicine Phoenix, Phoenix, AZ USA

## Abstract

**Background:**

Different PET tracers can be used to measure neuritic amyloid plaque deposition in human brain. While mean cortical‐to‐cerebellar standard uptake value ratios (SUVRs) generated using different radiotracer methods can be transformed into Centiloid measurements to facilitate comparisons among the resulting amyloid plaque measurements, the level of agreement as measured by the strength of the correlation between the measurements does not change. In this study, paired 18F‐labeled (i.e., florbetapir, florbetaben, flutemetamol, and NAV4694) and 11C PiB radiotracers from the same research participants to test the hypothesis that the correlation between amyloid plaque measurements derived using different radiotracers can be improved by accounting for variability in white matter uptake.

**Method:**

For all four 18F‐labeled radiotracers, paired 18F‐radiotracer and PiB‐derived PET images were downloaded from the Centiloid project (www.gaain.org/centiloid‐project). An in‐house image‐analysis pipeline was used to generate mean cortical‐to‐cerebellar SUVRs (MC‐SUVRs) from every PET image. Two approaches to account for white matter contributions to MC‐SUVR in the inherently low‐resolution PET images: 1) a linear regression approach to account for white matter contributions using a FreeSurfer‐defined UnsegmentedWhiteMatter SUVR; and 2) a regional spread function (RSF)‐based partial volume correction (PVC) technique. Pearson’s correlation coefficient was used to assess the agreement between F18 tracer‐based measure and PIB. Steiger’s test was used to determine whether accounting for white matter signal significantly improves agreement.

**Result:**

Accounting for white matter signal improved the agreement between each 18F radiotracer‐based MC‐SUVR and the corresponding PiB‐derived MC‐SUVR. Using the regression approach, the correlations with PiB SUVRs were significantly improved for florbetapir (p<0.0001) and flutemetamol (p=0.03) but did not reach significance for florbetaben and NAV4694. For the PVC‐based approach, correlations with PiB SUVRs were significantly greater for all four 18F‐tracers (p<0.05).

**Conclusion:**

The relationship between mean cortical measurement of amyloid plaque burden using different PET tracers can be significantly improved by accounting for differences in white matter uptake. Additional studies are needed to optimize this approach and account for any other potential confounds.